# Hybrid Magnetic-DNA Directed Immobilisation Approach for Efficient Protein Capture and Detection on Microfluidic Platforms

**DOI:** 10.1038/s41598-017-00268-8

**Published:** 2017-03-15

**Authors:** Elaheh Esmaeili, Mohammad Adel Ghiass, Manouchehr Vossoughi, Masoud Soleimani

**Affiliations:** 1 Institute for nanoscience and nanotechnology, Sharif University of Technology, Tehran, 14588-89694 Iran; 2 Stem Cell Technology Research Centre, Tehran, 19977-75555 Iran; 3 Chemical and Petroleum Engineering Department, Sharif University of Technology, Tehran, 14588-89694 Iran; 4 School of Medical Sciences, Tarbiat Modares University, Tehran, 14115-116 Iran

## Abstract

In this study, a hybrid magnetic-DNA directed immobilisation approach is presented to enhance protein capture and detection on a microfluidic platform. DNA-modified magnetic nanoparticles are added in a solution to capture fluorescently labelled immunocomplexes to be detected optically. A magnetic set-up composed of cubic permanent magnets and a microchannel was designed and implemented based on finite element analysis results to efficiently concentrate the nanoparticles only over a defined area of the microchannel as the sensing zone. This in turn, led to the fluorescence emission localisation and the searching area reduction. Also, compared to processes in which the immunocomplex is formed directly on the surface, the proposed approach provides a lower steric hindrance, higher mass transfer, lower equilibrium time, and more surface concentration of the captured targets leading to a faster and more sensitive detection. As a proof-of-concept, the set-up is capable of detecting prostate-specific membrane antigen with concentrations down to 0.7 nM. Our findings suggest that the approach holds a great promise for applications in clinical assays and disease diagnosis.

## Introduction

The analysis of proteins with high sensitivity and specificity is critical for the early disease diagnosis and is the key factor for monitoring disease recurrence and therapeutic efficacy^1, 2^. Enzyme-linked immunosorbent assay (ELISA) is a traditional method progressively used to recognise the occurrence of a substance in a fluid specimen. Most ELISA tests have disadvantages in terms of handling and analysis time, sample and reagent consumption, as well as automation and portability capabilities^3^. Moreover, the interactions between the involved components in these heterogeneous surface-based assays that recognise analytes in the solution, depend on factors including the surface concentration of the binding sites, concentration and diffusion constant of the targets in the solution, and binding affinity of probes for their respective targets^4^. In these platforms, reduction of the species near the surface upon binding to it and the subsequent diffusion of them towards the surface can lead to suboptimal detection limits and longer incubation times. In contrast to the traditional solid substrates such as ELISA plates, the semi-homogenous suspension of nanoparticles functionalised with capture antibodies as the mobile substrates endue them with rapid reaction kinetics and better detection sensitivities^5, 6^. Also, approaches to concentrate the immunocomplex on the surface for detection, such as applying a force to the species to get them close to the surface are noteworthy^7, 8^.

Magnetic nanoparticles (MNPs) possess impressive merits including a large surface to volume ratio, low cost of synthesis, short analysis time, magnetic susceptibility, low toxicity, and compatibility with biomaterials. This makes them appropriate for a wide variety of applications including biosensing^9–12^, drug delivery^13–15^, and sample purification^16–18^. The usage of MNPs as biomolecule carriers is promising, since the biomolecule confers the specificity of the MNP assemblies towards the targeted molecules and they can be manipulated by external magnetic fields^19–22^. Thus, the matrix effects are successfully addressed by the improved washing steps eliminating the need for sample pretreatments using centrifugation or chromatography. Compared to the diffusion-limited immobilisation methods, the assemblies are directed towards the defined imaging zone to reduce the searching area and enhance the surface concentration of the captured target^23, 24^.

Immunoassays offer high specificity due to the use of antibodies against the analyte of interest. However, the surface immobilisation of such antibodies can challenge their integrity, activity, stability, and specificity, thus lowering the sensor performance and promptness^25, 26^. To address these issues, the DNA-directed immobilisation (DDI) is a proper candidate to localise proteins and antibodies^27–29^. In DDI, an antibody molecule tailed with ssDNA is assembled onto the surface by hybridisation with the complementary ssDNA probe recognising the antigen specifically. This kind of immobilisation has several advantages over the direct covalent attachment of antibodies. It increases the availability of the binding sites for analyte capture, as the reduced steric hindrance allows more favourable orientations for binding. In addition, this kind of immobilisation provides the ability to reprogram the sensor surface using different sets of antibodies conjugated to the same DNA sequences, and surface renascence by de-hybridisation of the antibody-DNA conjugates^30^.

In recent years, MNPs-based immunoassays have been adapted to the lab-on-a-chip/microfluidic format for pathogen detection^31, 32^. Microfluidic technology allows the miniaturisation of devices, which results in a minimum consumption and processing of sample and reagents (microliters to nanoliters) and minimum chemical waste, shorter analysis time, portability, and lower detection limits (LODs)^33–35^. Such devices can be advantageously utilised for point-of-care diagnostics, where they provide potentially fast and low-cost analyte detection.

Handling of MNPs in a microfluidic channel using magnetic fields is an efficient and prevalent technique for diverse chemical and biological applications including magnetic separation^36, 37^ and mixing^38, 39^. Magnetism-based microsystems can be classified based on whether the magnetic field actuation is integrated into the device or not. Active magnetic microsystems use on-chip micro-electromagnets that can be addressed separately^40^. Joule heating effect due to the relatively high current densities, complex processes for the integration of the micro-fabricated magnets into the microfluidic devices and the limited field strength (0–100 mT) are the drawbacks of such systems^41, 42^. On the other hand, off-chip electromagnets or permanent magnets are utilised in passive magnetic microsystems. This results in a simple operation, lower cost, no unwanted heat generation, and larger magnetic fields (>0.5T) and forces^40, 42^. The fields produced by the Off-chip permanent magnets can be significantly improved by tuning the parameters including magnetic material, geometry and configuration of the magnets.

In this study, we describe the development of a microfluidic immunofluorescence system based on a hybrid magnetic-DNA directed immobilisation approach for the selective detection of Human Glutamate Carboxypeptidase II, also known as prostate-specific membrane antigen, PSMA. PSMA is a 750 residue, 100 kDa glycoprotein that is overexpressed on the surface of prostate cancer cells and has been well-characterised as a candidate for prostate cancer diagnosis^43^. PSMA is elevated from 200–300 ngmL^−1^ (2–3 nM) in healthy patients to 300–650 ngmL^−1^ (3–6.5 nM) in patients with prostate cancer^44^.

**Figure 1:**
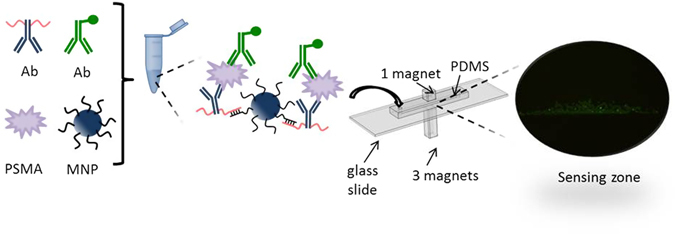
Schematic representation of the immunocomplex preparation in solution using DDI method. The sample containing the immunocomplex composed of a MNP, anti-PSMA antibody-functionalised with ssDNA molecules, antigen, and AlexaFluor-488 conjugated secondary antibody is then introduced into a magnetic configuration including a microchannel and gets concentrated on the sensing zone.

We synthesised MNPs linked to antibodies via a robust hybridisation between a DNA tether attached to the antibody and its complementary sequence immobilised on the MNPs. These assemblies are dispersed into a solution to capture their antigen counterparts and the secondary AlexaFluor-488 conjugated antibody as schematically shown in Fig. 1. A microchannel is filled with the sample followed by the optical analysis when there is no flow in it, static situation. To concentrate the immunocomplexes over the sensing zone at the centre of the microchannel, we designed and implemented a passive magnetic microsystem composed of two series of cubic permanent magnets situated above and below the microchannel. Finite element analysis was used to investigate the magnetic configuration design considering the number of the magnets on the top and bottom of the structure and the asymmetry. The results were utilised to improve the way the magnetic force is exerted on the MNPs and to form an efficient sensing zone for optical detection of the target.

## Numerical simulations

In the vicinity of a permanent magnet, the magnetic flux density is expressed by:

1 $$B={\mu }_{0}(H+M)$$

where *μ*_0_ is the permeability of free space, *H* is the magnetic field, and *M* is the magnetisation due to the permanent magnet^45^. A force is exerted on a constant magnetic dipole moment, *m*, and can be expressed as a function of the magnetic potential energy, *U*, as:

2 $${F}_{m}=-{\nabla }(U)={\nabla }(m\cdot B)\approx (m\cdot {\nabla })B$$

The magnetic dipole moment of a superparamagnetic particle surrounded by a non-magnetic medium is expressed by:

3 $$m=V{\rm{\Delta }}\chi H$$

where *V* is the volume of the particle and Δ*χ* is the difference in the magnetic susceptibilities of the particle and medium. Using Eqs (1) and (3), the magnetic force exerted on the particle can be expressed by:

4 $${F}_{m}=\frac{V{\rm{\Delta }}\chi }{{\mu }_{0}}(B\cdot {\nabla })B$$

For a given particle, *F*_*m*_ can be calculated if we know (*B*·*∇*)*B* at a point in space.

In 3D modelling, 3 components of *F*_*m*_ can be expressed by:

5 $$\{\begin{matrix}{F}_{x}=\frac{V{\rm{\Delta }}\chi }{{\mu }_{0}}({B}_{x}\frac{\partial {B}_{x}}{\partial x}+{B}_{y}\frac{\partial {B}_{x}}{\partial y}+{B}_{z}\frac{\partial {B}_{x}}{\partial z})\\ {F}_{y}=\frac{V{\rm{\Delta }}\chi }{{\mu }_{0}}({B}_{x}\frac{\partial {B}_{y}}{\partial x}+{B}_{y}\frac{\partial {B}_{y}}{\partial y}+{B}_{z}\frac{\partial {B}_{y}}{\partial z})\\ {F}_{z}=\frac{V{\rm{\Delta }}\chi }{{\mu }_{0}}({B}_{x}\frac{\partial {B}_{z}}{\partial x}+{B}_{y}\frac{\partial {B}_{z}}{\partial y}+{B}_{z}\frac{\partial {B}_{z}}{\partial z})\end{matrix}$$

In this work, we use a magnetic configuration composed of cubic permanent magnets of 5 × 5 × 5 mm^3^ which are of 1.3T in remanent flux density. Any number of the cubes can be stuck to each other to form a bar magnet and modify the magnetic properties. The magnets are situated at the centre along *x* and *y* axis (*x* = 0 and *y* = 0) with magnetisation along *z* axis. The magnets can be positioned over or under the chip and different results are expected also depending on the number of magnets. As shown schematically in Fig. 1, the configuration includes the glass slide as the substrate, the PDMS slab containing the 3 cm-long microchannel, and the magnets over the PDMS slab and under the substrate positioned at the channel centre.

The magnetic set-up can be configured in three different ways. One bar magnet can be put under the substrate. Two bar magnets of different height can be put so that the same poles or counter poles are facing each other. Gassner and her group studied these three cases with bar magnets of the same height and showed that the superparamagnetic particles can be concentrated at one point for attraction mode and two points for the repulsion mode, demonstrating the preference of the attraction mode^46^. However, they only took into account the situation in the close vicinity of the magnets where both magnets were of the same height, symmetric configuration.

Different magnetic configurations are defined by the number of magnets on the top, a, and under the substrate, b, named as a-b. We studied the effect of different magnetic configurations on the magnetic force distribution within the microchannel. In the model, Gauss’ Law for the magnetic field was solved using the scalar magnetic potential as the dependent variable. For the configuration to be efficient, the magnetic force exerted on the MNPs should direct them towards the sensing zone along the microchannel and concentrate them on the surface.

## Results and Discussion

**Figure 2:**
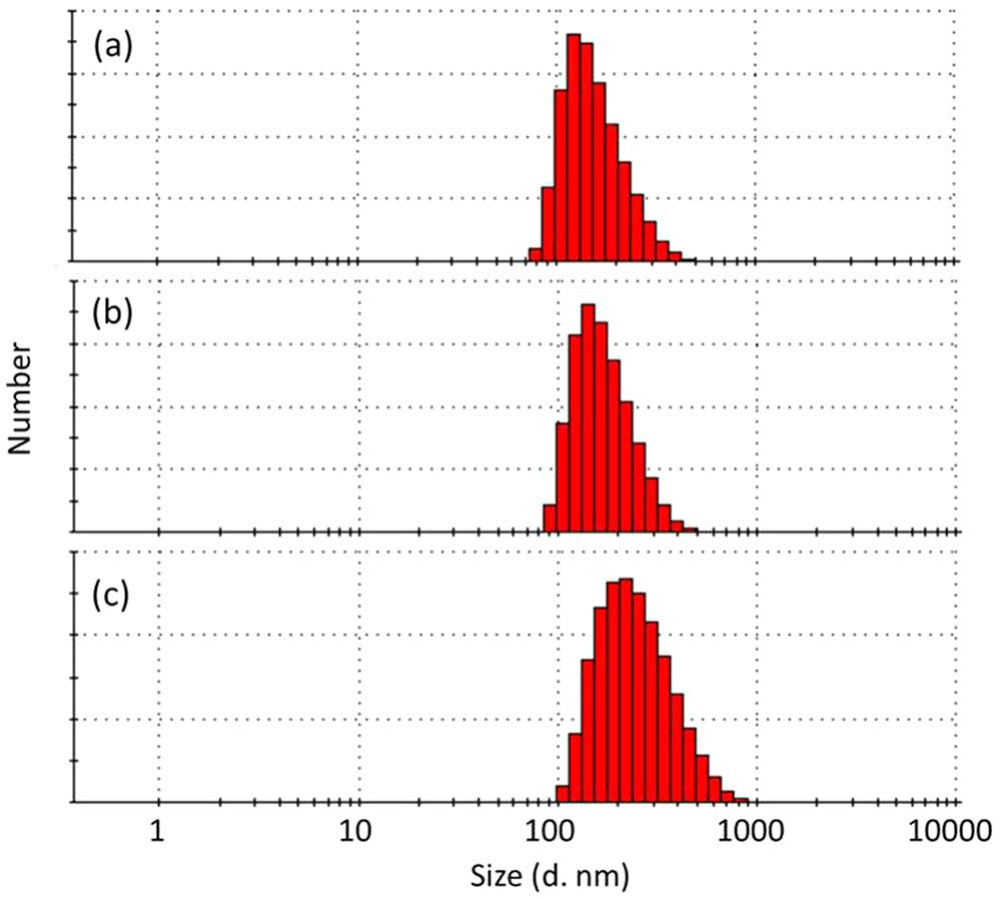
Hydrodynamic diameter distribution of the MNPs determined by DLS measurements: (**a**) streptavidin coated MNPs (**b**) ssDNA conjugated MNPs, and (**c**) anti-PSMA antibody conjugated MNPs.

The ssDNA conjugation to streptavidin coated MNPs and antibody coating on ssDNA conjugated MNPs were characterised with dynamic light scattering (DLS) that measures the hydrodynamic diameter of the MNPs in their dispersion state. The DLS measurement results in the hybridization buffer are shown in Fig. 2. It can be seen that before conjugation, streptavidin coated MNPs have an overall size around 122 nm. After modification with ssDNA and thereafter ssDNA conjugated antibody over ssDNA conjugated MNPs, the particles increased in size to around 142 and 190 nm, respectively, indicating the ssDNA and antibody biomolecules are incorporated in the surface of MNPs.

**Figure 3:**
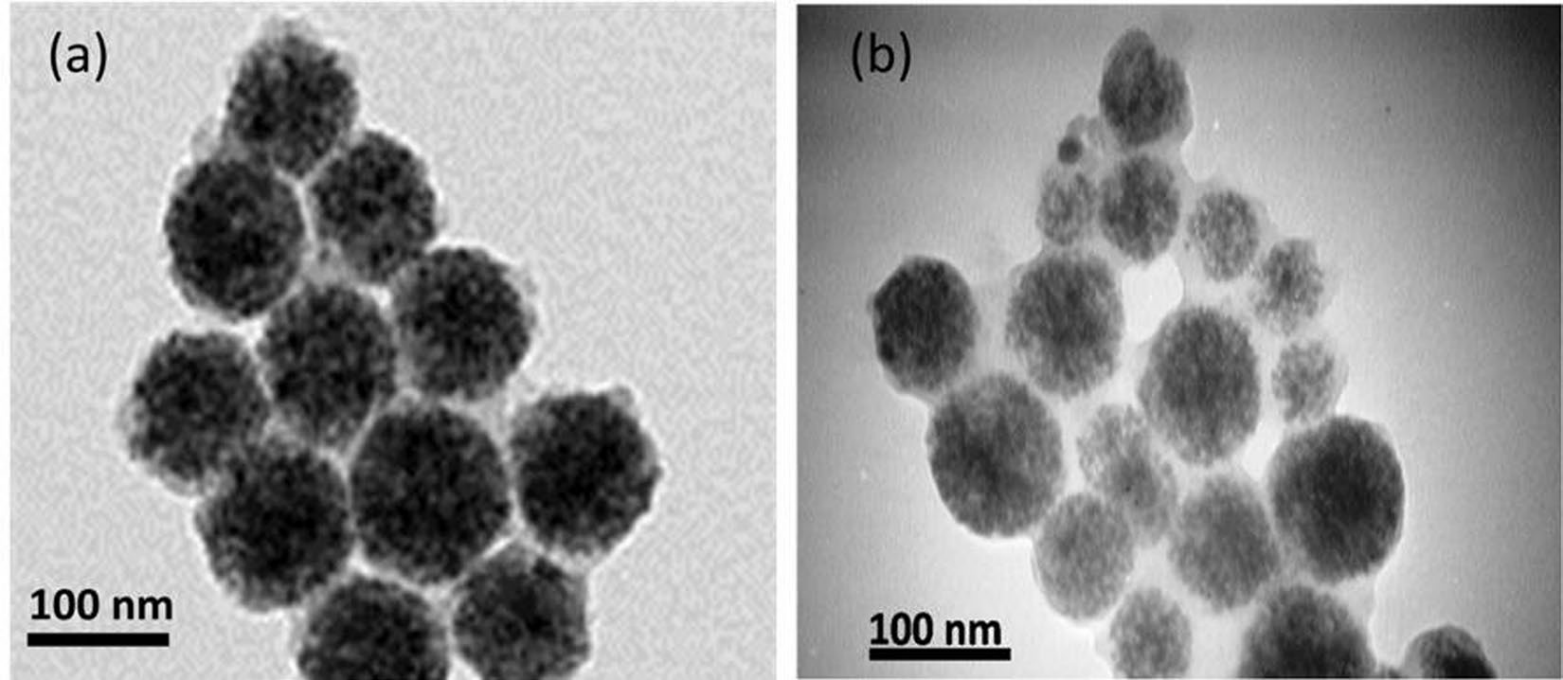
TEM images of the (**a**) streptavidin coated MNPs and (**b**) antibody conjugated MNPs.

Figure 3 shows the TEM images of the streptavidin coated MNPs (provided by Ademtech) and antibody conjugated MNPs. The average core size is about 100 nm, but the shell thickness in the antibody conjugated MNPs increased comparing to streptavidin coated MNPs due to the conjugation of antibodies to MNPs.

The surface charges of streptavidin, ssDNA and antibody coated MNPs were determined by zeta potential measurement. The zeta potential of the initial streptavidin coated MNPs was −8.9 mV owing to the presence of streptavidin on the surface of nanoparticles. After ssDNA conjugation, zeta potential substantially increased to −33.2 mV due to the exposed phosphate groups of ssDNA. Hybridization of ssDNA with ssDNA-antibody screens some negatively charged phosphate groups, decreasing the zeta potential to −28.4 mV.

**Figure 4:**
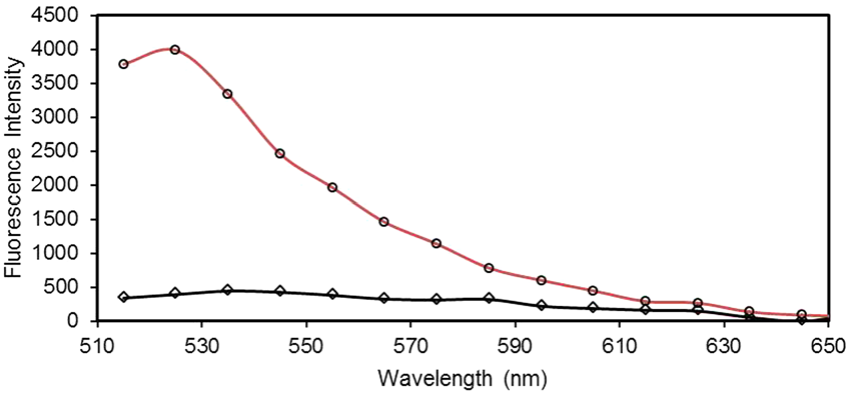
Comparison of the fluorescence intensities recorded for the immunocomplexes hybridised to the ssDNA probes immobilised on the streptavidin coated surface of the microtiter plate heterogeneously (rhombi) and that of the MNPs semi-homogenously (circles).

Figure 4 compares the recorded fluorescence intensity of the immunocomplexes hybridised to the ssDNA probes immobilised on the streptavidin coated surface of the microtiter plate and that of the MNPs. The latter case leads to a 10-fold increase in the intensity verifying the significant effect of the steric hindrance reduction and the mass transport improvement. The achieved intensity enhancement is of importance to improve the detection limit of the point-of-care applications.

The merits of using a microchannel including the lower consumption of samples and expensive reagents, portability and lower detection limits motivated us to perform the immunofluorescence experiments in a microchannel. The MNPs should be concentrated on a defined region of the microchannel to reduce the searching area by enhancing the surface localisation of the captured targets and consequently facilitating the fluorescence emission recording. The results of the numerical analysis carried out to evaluate the magnetic force acting on the MNPs produced by the cubic permanent magnets set up in different configurations are discussed here.

The magnetic particles in the channel are confined in *y* and *z* directions to the width and height of the microchannel, respectively. Thus, our main concern is what happens to them in the *x* direction. Though, it is of interest to concentrate them on the bottom surface of the microchannel.

**Figure 5:**
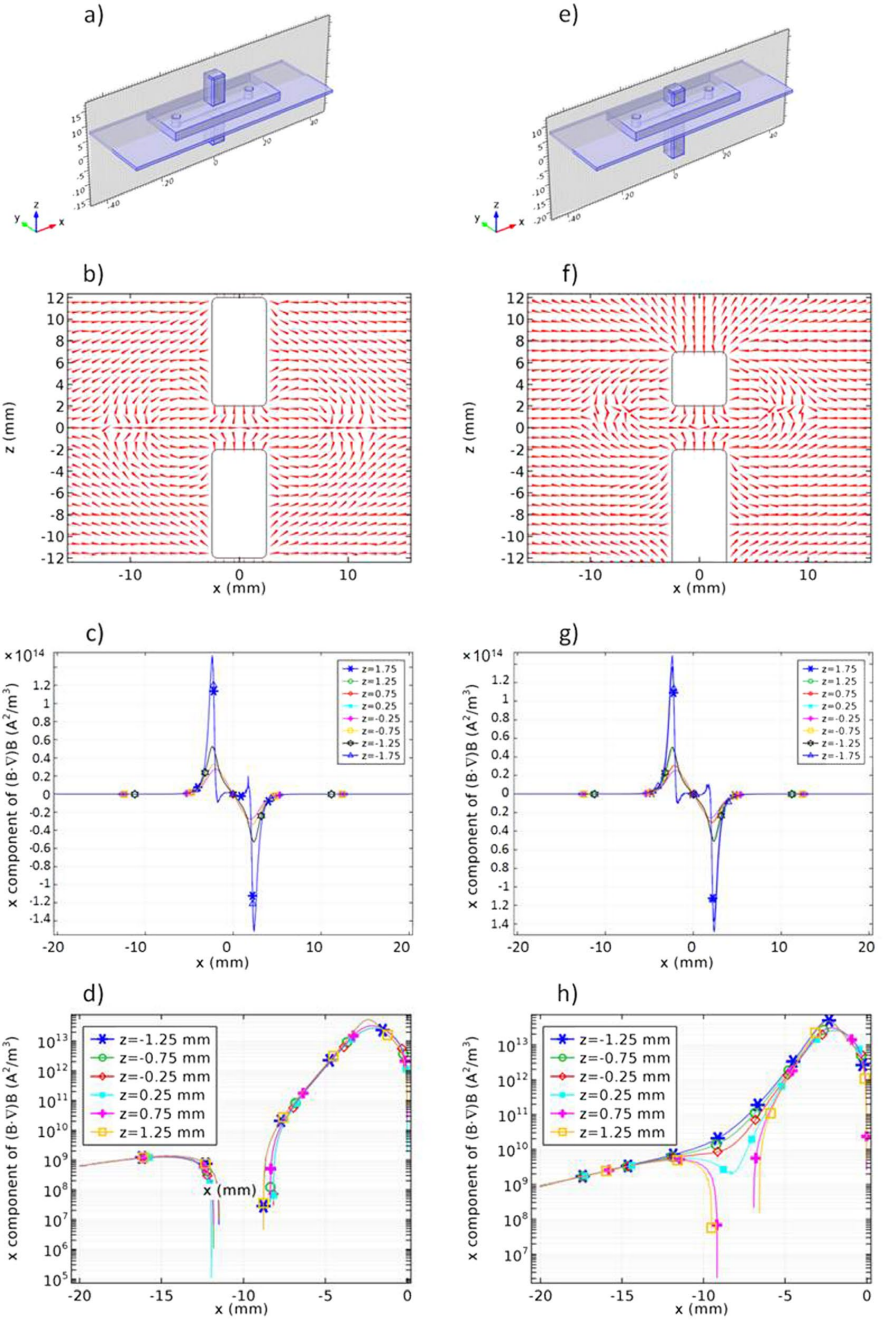
(**a**) schematic representation, (**b**) Normalised force vectors in the cross-sectional *xz* plane, (**c** and **d**) the *x* component of (*B*·*∇*)*B* for 2–2 symmetric magnetic configurations at *z* = −1.75 to 1.75 mm with 0.5 mm steps, (**e**) schematic representation (**f**) Normalised force vectors in the cross-sectional *xz* plane, (**g** and **h**) the *x* component of (*B*·*∇*)*B* for 1–3 asymmetric magnetic configurations at *z* = −1.75 to 1.75 mm with 0.5 mm steps. All dimensions are in mm.

Figure 5b and f compares the vector representation form of *F*_*m*_ in symmetric (2–2) and asymmetric (1–3) configurations as shown schematically in Fig. 5a and e, respectively. No particle can stay at the points where *F*_*m*_vectors diverge. On the other hand, particles tend to aggregate around the points where *F*_*m*_ vectors converge, since the magnetic force directs them towards that point. The latter condition is of importance in our application. In the asymmetric configuration, the aforementioned regions are shifted in *z* direction towards the smaller magnet. Thus, if the channel is situated close enough to the larger magnet; no zero force regions are formed along the channel other than the central one.

To study the different configurations in a quantitative manner, *x* component of (*B*·*∇*)*B* was extracted from the simulation results at different *z* values. Figure 5c and g show the *x* component of (*B*·*∇*)*B* for the 2–2 symmetric and 1–3 asymmetric configurations at *z* = −1.75 to 1.75 mm with 0.5 mm steps. At *z* = 0 mm, one obvious zero is seen at *x* = 0 mm. For *x* > 0, negative values of *x* component of (*B*·*∇*)*B*, a negative force is exerted on a superparamagnetic particle on the right hand side of the channel attracting it towards the centre and for *x* < 0, positive values of *x* component of (*B*·*∇*)*B*, a positive force is exerted on a superparamagnetic particle on the left hand side of the channel attracting it towards the centre. The curves contain two extrema except for the cases where *z* is close enough to either top or bottom magnets. For instance, at *z* = −1.75 mm and *z* = 1.75 mm two additional extrema emerges close to the vertical edges of the magnets.

Figure 5d shows the *x* component of (*B*·*∇*)*B* for the 2–2 symmetric configuration at *z* = −1.75 to 1.75 mm with 0.5 mm steps in logarithmic scale. More zeros are observable in logarithmic scale around *x* = −10 mm. Due to the structural symmetry with respect to the *yz* plane; more zeros are expected around *x* = 10 mm, similarly. Those are unwanted concentration regions along the microchannel.

Figure 5h shows the *x* component of (*B*·*∇*)*B* for the 1–3 asymmetric configuration at *z* = −1.75 to 1.75 mm with 0.5 mm steps in logarithmic scale. In this case, no unwanted zero emerges for *z* < 0 or in the bottom half of the gap between the magnets. Therefore, the asymmetric configuration is appropriate to concentrate the superparamagnetic particles at the centre of the microchannel where there is the only one zero for *F*_*x*_ in the bottom half of the gap, close to the larger magnet. This is the proper z range for the microchannel.

**Figure 6:**
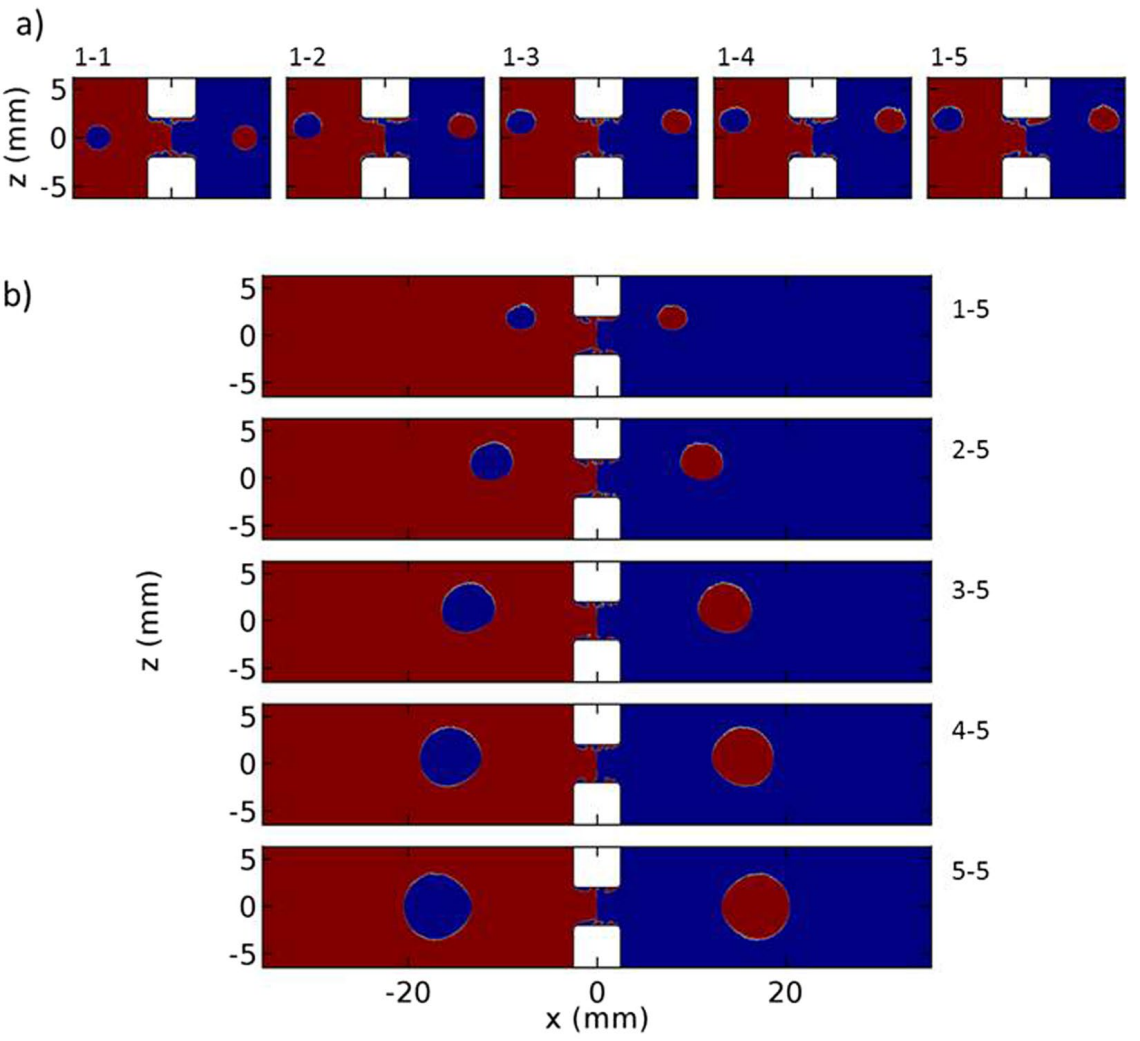
Dependence of the size and position of the elliptical regions of opposite *F*_*x*_ on the magnetic configuration asymmetry for cases of (**a**) 1–1, 1–2, 1–3, 1–4, and 1–5 and (**b**) 1–5, 2–5, 3–5, 4–5, and 5–5.

Figure 6 shows the effect of asymmetry in the magnetic configuration on *F*_*x*_. To minimise the number of the cubic magnets used in the configuration, we put one magnet on the top, *a* = 1, and adjusted the number of magnets at the bottom, *b* = 1 to 5, Fig. 6a. In Fig. 6 rightwards and leftwards *F*_*x*_ at each point are illustrated by red and blue, respectively. For all configurations, the blue area for *x* > 0 meets the red area for *x* < 0 at the centre where the particles are expected to get concentrated. Also, elliptical regions of opposite force are seen on both regions. Their size and position depend on the amount of asymmetry. For these regions, converging *Fx* is observed on a portion of the border. That means the particles are concentrated at the intersections of the microchannel and those borders, leading to the formation of unwanted concentration regions. If the elliptical regions are situated above or below the microchannel, those intersections can be avoided and consequently no unwanted concentration region emerges. This can be done by adjusting the configuration including the magnets and microchannel position.

For 1–1 configuration, 50%, the centre of the elliptical regions are exactly situated at *z* = 0. By increasing the asymmetry up to 1–3 configuration, 75%, the regions are shifted upwards and they are situated above *z* = 0. The shift is negligible for further increase in the asymmetry. According to these results, the microchannel can be situated below *z* = 0 to form the unique concentration region at the centre. We chose *z* = −1 mm for the bottom surface of the microchannel. It should be noted that no significant change in the size and distance of the elliptical regions along *x* axis is observable for these configurations.

Figure 6b compares the situation of the elliptical regions of the opposite *F*_*x*_ for the cases where *b* = 5 and *a* = 1 to 5. The size and distance of the regions along *x* axis increases, as *a* increases. It is concluded that in an asymmetric configuration, the height of the smaller magnet dominantly affects the size and distance of the regions along *x* axis.

**Figure 7:**
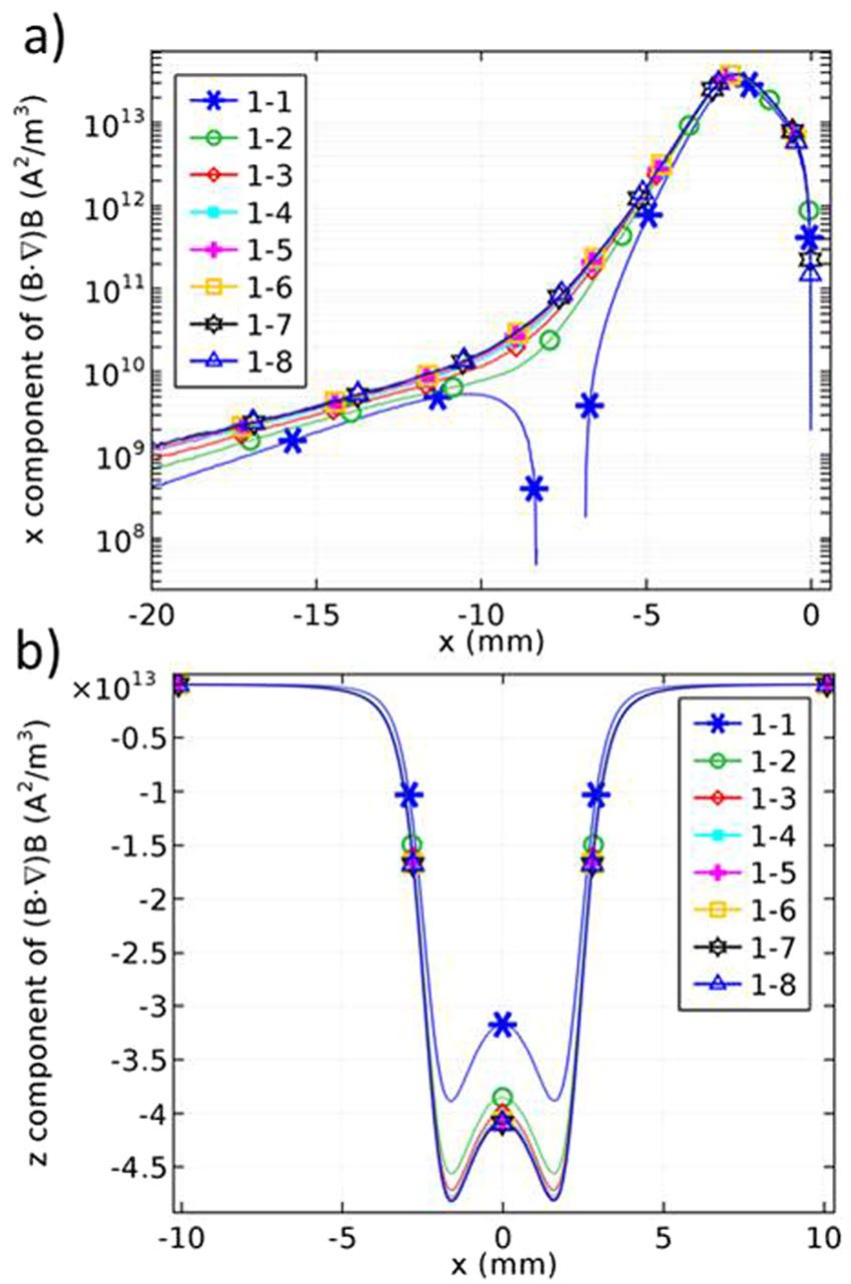
(**a**) *x* component and (**b**) *z* component of (*B*·*∇*)*B* for *a* = 1 and *b* = 1 to 8 at *z* = −1 mm along the microchannel.

Figure 7a shows the *x* component of (*B*·*∇*)*B* for *a* = 1 and *b* = 1 to 8 at *z* = −1 mm, the bottom surface of the microchannel. The magnitude of the *x* component of (*B*·*∇*)*B* increases as *b* increases. However, the increase is negligible for *b* > 3. Moreover, the magnitude of the *z* component of (*B*·*∇*)*B* follows the similar trend as seen in Fig. 7b. Based on these results, we chose 1–3 magnetic configuration for the next steps of the study. The magnitude of the force exerted on the particles affects the time required to achieve the appropriate concentration on the sensing zone. The details of this aspect are beyond the scope of this work.

**Figure 8:**
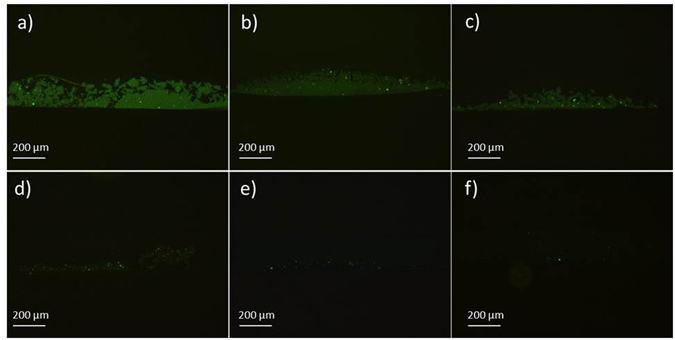
Fluorescence images of the concentrated immunocomplexes on the sensing zone of 1–3 set-up for samples of (**a**) 20 nM, (**b**) 10 nM, (**c**) 5 nM, (**d**) 3 nM, (**e**) 1.5 nM and (**f**) 0.7 nM PSMA protein concentrations in the buffer solution.

**Figure 9:**
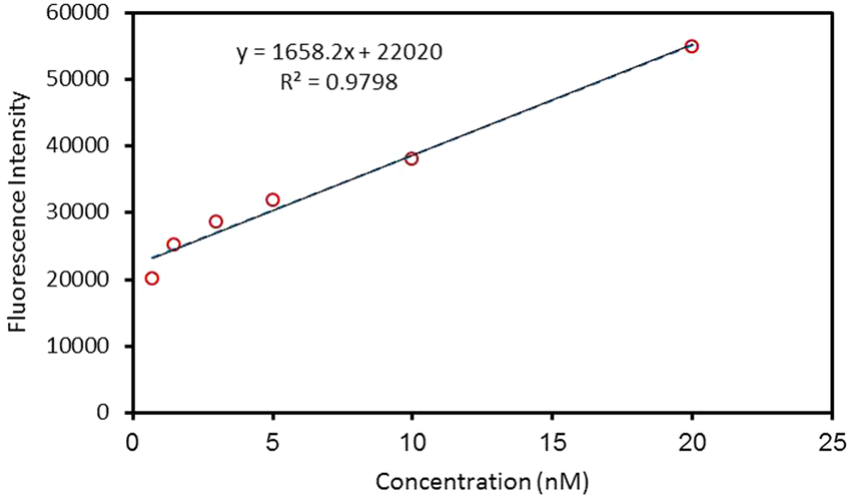
The response of the assay for PSMA antigen in the range of 0.7–20 nM, showing the detection limit of 0.7 nM.

We evaluated the detection limit of the set-up composed of 4 cubic magnets, 1–3 asymmetric magnetic configuration, in a qualitative manner using different PSMA protein dilutions in the range of 0.7–20 nM. Figure 8a–f shows the images of the concentrated immunocomplexes on the sensing zone for samples of 0.7, 1.5, 3, 5, 10, and 20 nM PSMA proteins. The images were taken at a fixed exposure and the pixel intensities were used to perform a quantitative analysis using Image J software as shown in Fig. 9. The concentrations of down to 0.7 nM can be observed using the fluorescence microscope. This value is lower than the critical threshold of the prostate cancer risk, 3 nM.

**Figure 10:**
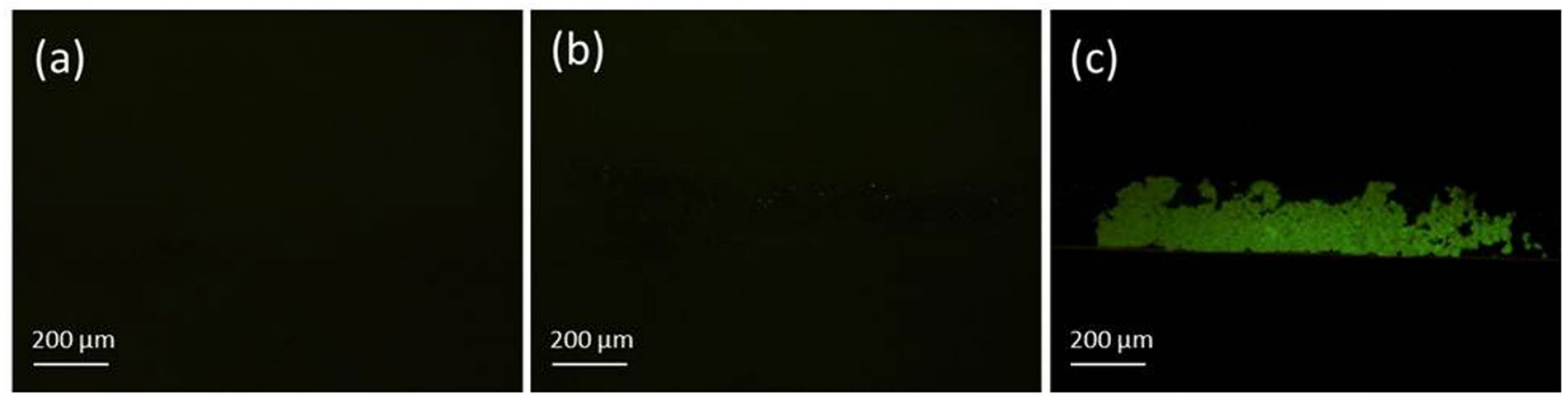
Fluorescence images of the concentrated immunocomplexes on the sensing zone of 1–3 set-up for serum samples of (**a**) 0 nM, (**b**) 1 nM, and (**c**) 20 nM PSMA concentrations verifying the selectivity towards PSMA.

The selectivity of the introduced approach was also examined using serum samples of 0, 1, and 20 nM PSMA concentrations. As shown in Fig. 10a–c, the set-up provides a good selectivity towards PSMA, the target protein, where there are different types of other proteins in the serum. The results verify that no false statement was generated utilising the two monoclonal antibodies specific to PSMA antigen.

## Conclusions

In summary, we provide a hybrid magnetic-DNA directed immobilisation approach to enhance protein capture and detection within microfluidic platforms. Finite element analysis was used to study the magnetic configuration design and improve it. The influence of asymmetry on defining the efficient sensing zone was investigated and an appropriate magnetic set-up composed of 4 cubic magnets and a microchannel was implemented. The experimental results are also promising and indicate that the usage of magnetic nanoparticels helps to overcome the common mass transport limitation of surface-based detections, since the reaction of components is performed semi-homogeneously. As a proof-of-concept prostate-specific membrane antigen with concentrations down to 0.7 nM was detected successfully using the implemented set-up. The proposed approach providing a suitable detection limit and good selectivity can be utilised in clinical assays and disease diagnosis systems. The achievements pave the way for a new generation of magnetic nanoparticle-based biosensors.

## Experimental

### Materials

We utilised the PSMA-specific monoclonal antibodies (D2B^47^ and 2G7^48^) generated in mouse, its antigen (SLIN tagged GCPII (44–750)), 100 nm Streptavidin coated MNPs (purchased from Ademtech) and biotin and amine functionalised DNA oligonucleotides (purchased from Integrated DNA Technologies, HPLC purity). Streptavidin coated microtiter plates and AlexaFluor® 488 Monoclonal Antibody Labeling Kit were obtained from ThermoFisher Scientific. Antibody-oligonucleotide conjugation kit was purchased from SoluLink (San Diego, CA). Sylgard 184 poly (dimethylsiloxane) (PDMS) and its curing agent were bought from Dow Corning. Negative photoresist, SU-8 2075 was purchased from Microchem. Cubic, Neodymium magnets, 5 × 5 × 5 mm, were purchased from supermagnete, Germany to concentrate MNPs on the surface. All other chemicals were obtained from Sigma-Aldrich.

The size distribution and zeta-potential of each formulation were determined with dynamic light scattering (DLS) (Malvern Zetasizer Nano ZS, UK). The fluorescence images were recorded with a fluorescence microscope (Nikon, Eclipse TE2000-S, Japan) and the fluorescence intensity in a 96-well microtiter plate was measured using a fluorescence plate reader (Cytation 3, Biotek, USA). The antibody conjugated MNPs were imaged using a Zeiss-EM10C Transmission Electron Microscope (TEM) operated at 80 kV accelerating voltage. The specimen of TEM was prepared by placing 1 mg/ml aqueous suspension of antibody conjugated MNPs on the carbon coated copper grids.

### Fabrication of the microchannel

The microchannel was fabricated using polydimethylsiloxane (PDMS) by soft lithography technique. For this purpose, the SU-8 photoresist was spin coated on the silicon wafer to achieve the desired thickness. After soft baking to evaporate the solvent and densify the film, the substrate was exposed to UV light through the photomask consisted of a 200 µm-wide and 3 cm-long microchannel and post baked. Finally, the master mould was formed by wet etching. Afterwards, PDMS pre-polymer and its curing agent were mixed in a 10:1 ratio (w/w), poured on the mould and cured (80 °C, 1 hour). The PDMS slab was released from the mould and the inlet and outlet ports were punched into it. It was the bonded onto a glass slide to form the microchannel.

### DNA-antibody conjugation

To create a DNA-labelled antibody, antibody and oligos must be completely desalted and buffer exchanged into pH 8.0 phosphate buffered saline (PBS) using Zeba^TM^ desalt spin columns. Succinimidyl-4-formylbenzamide (S-4FB) in Dimethylformamide (DMF) was added to the 5’aminated oligomers (NH_2_-CAA AAC AGC AGC AAT CCA ATG CGC AGA CAC CCG ATT ACA AAT GC) in PBS and incubated at room temperature for 2 hours to allow the reagents to react with the amino-oligo. Separately, succinimidyl-6-hydrazino-nicotinamide (S-HyNic) in DMF was added to 2.8 mg/ml of antibodies at a molar ratio of 15-fold of S-HyNic to the antibody. Excess S-HyNic and S-4FB were removed and samples were buffer exchanged into pH 6.0 PBS using Zeba^TM^ column. Derivatised DNA and antibodies were then combined and allowed to react at room temperature for 2 hours and desalted in PBS using Zeba^TM^ column.

### Synthesis of ssDNA conjugated surface

The immobilisation of ssDNA on the surface was performed using the streptavidin coated surface, either MNPs or microtiter plate, and biotin-conjugated oligonucleotide, 5′-biotin- G CAT TTG TAA TCG GGT GTC TGC GCA TTG GAT. For the synthesis of ssDNA conjugated MNPs, the reaction mixture consisted of 0.5 mg/ml of streptavidin coated MNPs, biotinylated DNA oligonucleotides, 5 mM-Tris, 0.5 mM-EDTA, 1 M-NaCl and 0.05% tween 20 was prepared and incubated for 30 min at 37 °C. The product was then rinsed twice with Tris-buffered saline, 25 mM-Tris, 150 mM-NaCl, 0.1% BSA, 0.05% Tween-20, pH 7.2, to remove any impurities. For comparison, the ssDNA conjugation on the surface of the streptavidin coated microtiter plate was performed similarly without MNPs.

### Immunoassay experiment

To compare the semi-homogenous and heterogeneous immunoassay methods, we performed the experiments on the streptavidin coated MNPs and the streptavidin coated 96-well microtiter plates, respectively. For the semi-homogenous method, the ssDNA conjugated MNPs, ssDNA conjugated antibodies to be linked to the magnetic beads, PSMA antigen, and AlexaFluor-488 conjugated 2G7 antibodies were mixed in a microtube and incubated at 37 °C for 3 hours. After washing, the products were transferred to a 96-well microtiter plate and the fluorescence intensity was measured using a fluorescence plate reader. For the heterogeneous method, the procedure was similar except that the components were mixed and incubated in a 96-well microtiter plate coated with streptavidin and the conjugated ssDNA instead of using the ssDNA conjugated MNPs.

For the immunoassay experiments in the microchannel, the components including the ssDNA conjugated MNPs were mixed and incubated at 37 °C. The complex was then magnetically washed and concentrated over the sensing zone of the microchannel using the designed magnetic configuration. Afterwards, the fluorescence emission was recorded with a fluorescence microscope.

It should be mentioned that the serum sample was prepared with the approved protocol of ethics and with the consent of the donor.
